# Temporally parallel facilitation of same-colored objects beyond spatial selection

**DOI:** 10.1016/j.ynirp.2025.100302

**Published:** 2025-11-26

**Authors:** S. Wehle, C. Gundlach, M.M. Müller

**Affiliations:** Experimental Psychology and Methods, Universität Leipzig, Leipzig, Germany

**Keywords:** Visual attention, Attentional shifts, Feature processing, Steady-state visual evoked potentials (SSVEP), Electroencephalography (EEG), Spatial cueing, Object processing, Visual perception

## Abstract

In a probabilistic spatial cueing experiment, we cued one out of four objects/arcs that were arranged in a circle to test whether the cued arc/object would result in strictly object-based processing restricting facilitation of features to the cued object, or whether we observe global feature-based spread across object boundaries. Four arcs flickered at different frequencies, respectively, to evoke steady state visual evoked potentials (SSVEPs), allowing to investigate neural temporal dynamics in the early visual cortex following the presentation of the spatial cue. Initially, all arcs had identical colors and with spatial cue onset switched to a specific color configuration. In one configuration, one uncued arc had the same color as the cued one. We found global feature-based spread to same-colored elements across object boundaries in SSVEPs and behavioral responses. Once spatial attention was shifted to the cued location/arc, SSVEP amplitudes elicited by the cued arc and the same-colored uncued arc showed a temporally overlapping increase early after the spatial cue. Importantly, color processing was not required to perform the task, indicating that global feature-based spread occurs automatically, independent of task demands.

## Highlights

• Global feature-based processing occurs in a purely spatial attention task.
• Feature-based facilitation crosses object boundaries in early visual cortex.
• Task-irrelevant features are processed beyond the spatial focus of attention.
• Feature-based attention boosts detection at locations with matching features.

## 1 Introduction

The exact relationship between the three major attentional selection units - spatial, feature and object-based attention - is still under debate (Chapman and Störmer, 2024; Shomstein et al., 2023). Spatial selection refers to the idea that attention facilitates all information available at the attended location (Posner, 1980). Sensory gain was proposed as a neural mechanism for spatial attention, boosting sensory processing of stimuli at attended compared to unattended locations (Hillyard et al., 1998).

Feature-based attentional selection predicts that the visual system selects the relevant feature irrespective of its spatial location (M. M. Müller et al., 2006; Rossi and Paradiso, 1995; Treue and Martínez-Trujillo, 1999). First evidence for feature-based selection came from animal studies showing that neurons tuned to a certain feature exhibited enhanced activity if a stimulus with that feature is selected, even if that stimulus is located outside the receptive field of these neurons (Treue and Martínez-Trujillo, 1999). Evidence from human EEG studies showed that neural activity during feature-based selection at one location resulted in enhanced neural responses for stimuli located at an unattended location as well, again, demonstrating global spread of feature gain (Andersen et al., 2011; Forschack et al., 2017; Gundlach et al., 2023a, Gundlach et al., 2023b; M. M. Müller et al., 2018). A further relevant finding in the context of the present experiment concerns the obligatory nature of the global feature-based facilitation, even when it conflicts with the task at hand (Andersen et al., 2013).

In these previous studies, we recorded steady-state visual evoked potentials (SSVEPs; Norcia et al., 2015; Regan, 1989) by presenting stimuli in a frequency-tagged manner. SSVEPs have their generators in early visual cortex (Boylan et al., 2023; Di Russo et al., 2007; M. M. Müller et al., 1998a), and ongoing frequency-tagging offers an excellent tool to explore temporal neural dynamics of stimulus processing (de Lissa et al., 2020; Gundlach et al., 2020; M. M. Müller et al., 1998b), given the continuous oscillatory nature of SSVEPs. For instance, in a spatial selection experiment, Müller and colleagues (1998a) demonstrated that the time course of SSVEP amplitudes after the spatial cue mirrored the time course of behavioral responses to events at the cued location: Performance reached a higher level at an earlier time point when SSVEP amplitudes increased faster than when SSVEP amplitudes increased more slowly after the spatial cue.

Evidence for object-based attention came from studies revealing that behavioral responses to events outside the spatial focus are influenced by object borders (Egly et al., 1994; Francis and Thunell, 2022; Martínez et al., 2006). Egly and colleagues (1994) presented two rectangular objects in the periphery of the visual field. They cued one end of these rectangles exogenously and presented targets 300 ms later at either a cued (valid) or an uncued location (invalid). Notably, invalid events could either appear on the same or on a different rectangle compared to the cue. Response times to target events revealed costs, i.e., slower responses to invalid events as expected by spatial attention; however, costs were higher for events on a different object compared to the same object, demonstrating the same-object advantage (Chen, 2012). A decade earlier, Duncan (1984) demonstrated that discriminating between values of two features on the same object (e.g., the texture and the tilt of a line) was as accurate as discriminating between values of a single feature (e.g., only the texture of the line), whereas performance declined when features were on different objects (e.g., the texture of a line in one and the size of a box of the other object), even when both objects spatially overlapped (Cavanagh et al., 2023; Chen, 2012; Duncan, 1984). These studies demonstrated the relevance of objects as units of attentional selection.

In line with the above studies in object-based selection, the object integration account posits that the within-object advantage is explained by an automatic selection of all parts of an object when one part of that object becomes relevant (Davis et al., 2000; Davis and Driver, 1997; Egly et al., 1994; O'Craven et al., 1999). Evidence for this account revealed that the neural representation of the whole object (its features and its location) is subject to sensory gain modulation (Ekman et al., 2020; Ernst et al., 2013; Flevaris et al., 2013; Martínez et al., 2006, 2007; Roelfsema et al., 1998; Schoenfeld et al., 2014). For example, Schoenfeld and colleagues (2014) presented two spatially overlapping dot clouds whose elements were moving downwards at two different velocities. This formed two surfaces/objects, one colored, the other achromatic. Subjects were cued to the fast- or slow-moving surface and instructed to report transient horizontal displacements of elements of the cued surface. Event-related field potentials showed that the enhancement of cortical activity associated with processing the relevant feature (e.g., motion) was associated with facilitation of the uncued feature (e.g., color) of the same/relevant surface as well. Interestingly, the enhancement of cortical activity representing the processing of the relevant feature (e.g., motion) preceded the enhancement of the irrelevant feature of the relevant surface, indicating that object integration was accomplished in a two-stage process.

Feature- and object-based selection make different predictions about the selection of elements outside the focus of attention. While object-based theories postulate that features within an object are processed *conjointly*, feature-based accounts postulate that features are processed *independently* (Chapman and Störmer, 2021). Object-based theories imply that the selection of features is confined to object-boundaries (Duncan, 1984). Reading object-based selection strictly, this leads to the prediction that features should only be preferentially processed if they are a part of the object. Thus, object boundaries would limit the spread of feature processing. Feature-based selection, on the contrary, implies that the selection of features occurs in a spatially global manner. Consequently, feature-based selection should not be confined to object boundaries.

Contrary to the strict view of object-based selection, behavioral evidence indicated global feature effects across object boundaries, even if global feature spread is detrimental to the task (Chapman and Störmer, 2021), or if the feature whose global selection is in question was not task-relevant (Gonen et al., 2014). Gonen and colleagues presented four spatially separated arcs aligned in a circular pattern around the center of the screen. After a preview for 1 s, a small grey arc segment appeared on one of the objects, serving as a peripheral cue, non-informative of the upcoming target position. Targets appeared 200 ms later either at the cued location, at a different location on the same object, on a different (adjacent) object but equidistant to the cued location, or in the space between two objects (see Gonen et al., 2014, Experiment 2). Arcs sharing the same color were either located adjacent or opposite to each other. Thus, invalid targets could be presented on an arc in the same or a different color relative to the cued object. Results revealed effects of both object- and feature-based selection. Object-based selection was indicated by faster responses to events located on the cued object compared to a different object. However, the within-object advantage was not present if the different object was colored identical to the cued arc. That said, the detection of invalid targets on a different object was faster if this object was colored identically to the cued object, but slower if it was colored differently. Furthermore, the detection of events located in the space between two same-colored arcs was not facilitated. These results supported, on the one hand that feature-based facilitation was not bound to the cued object. On the other hand, object boundaries were maintained because a target in the space between two objects had no behavioral benefit. In line with these behavioral effects, there is also electrophysiological evidence for global feature spread across object boundaries (Adamian et al., 2020; Boehler et al., 2011).

A recent study suggested that global feature gain in early visual cortex needs time to propagate to the unattended location (Andersen and Hillyard, 2024). This study used a design from our previous studies by presenting two superimposed Random Dot Kinematograms (RDKs) with two different colors in the left and right visual hemifield, respectively, each flickering at a different frequency (Andersen et al., 2011). Before trial onset, subjects were cued to attend to one color at one side. Global feature gain was measured by the time course of SSVEP amplitude enhancement that was elicited by the RDK in the cued color at the uncued side. The authors reported a delay of about 150 ms, compared to the SSVEP amplitude enhancement at the cued side.

Contrary to that finding, in a very similar design, we reported that global feature gain occurred simultaneously at the to-be-ignored and the to-be-attended side (Gundlach et al., 2023b). These results showed that temporal dynamics of global feature-based spread are far from being clear. However, there are subtle differences between these two studies: For instance, in Andersen and Hillyard (2024), at the beginning of the trial, all RDKs were colored identically, and 800 ms after flicker onset, a color change occurred, while in our study, the two different colors were presented with trial onset. Further, in the first study, the cue was given before trial onset, while in our study, subjects were cued to one side and color about 1 s after trial onset. Another point that is important to mention: The RDKs that were used in these two studies are designed in a way that object grouping is not possible, thus results cannot automatically be transferred to a situation in which defined objects will be presented.

The present study was designed to fill two gaps. First, we aimed to test whether object or feature-based processing is the dominant process in a spatial selection (cueing) paradigm. The second aim was to uncover neural temporal dynamics of a possible feature spread across object boundaries in case of an automatic global feature-based facilitation. To achieve these objectives, we adapted the stimuli of Gonen and colleagues (2014) into an attentional shifting design. Each arc flickered at a unique frequency to obtain individual SSVEP amplitude time courses, respectively. Spatial selection was induced by a probabilistic spatial cue pointing to one of the four arcs. Then, target events could appear at either the cued (higher probability) or in one of the two adjacent arcs (lower probability). Subjects were instructed to respond as fast as possible to such a target regardless of its presentation location. Cueing one arc should either result in the processing of that arc/object and targets at an uncued arc should result in behavioral costs, regardless of its color. If, however, the processing of that cued arc that has a certain color triggers global feature-based color spread as well, then we would expect that targets at an uncued arc that shares the same color as the cued arc result in faster responses compared to when the uncued arc was in a different color.

Likewise, if object-based processing is an automatic process, we expect facilitation of the cued arc only, as measured by an increase in SSVEP amplitude elicited by the cued arc, but no difference in SSVEP amplitude between the uncued arcs presented in the same or a different color compared to the cued arc. If feature-based processing is the automatic process, SSVEP amplitudes should be higher for the uncued same-colored arc compared to the uncued differently-colored arc. If our data indicates global feature-based spread across object boundaries, analyzing the time course of SSVEP amplitude facilitation will allow us to test whether there is a temporal delay between facilitation of the cued and the uncued, same colored arc, like in Andersen and Hillyard (2024), or whether we replicate temporally parallel facilitation of both same-colored arcs (Gundlach et al., 2023b). Results demonstrated that global feature-based spread was not bound to object boundaries, and arcs that shared the same color as the cued arc were facilitated in a temporally parallel manner.

## *2* Methods

### *2.1* Participants

Sample size was determined a priori using G∗Power (Faul et al., 2009) for a power of 0.9 and an alpha level of 0.05, based on reported effects of feature-based enhancement of SSVEP amplitudes for stimuli in the periphery matching the color of the attended stimulus (*Min*_*d*_ = 0.66, *SD*_*d*_ = 0.25, see Brummerloh et al., 2019; Brummerloh and Müller, 2019; Müller et al., 2018). This yielded a sample size of 27. We recorded *N* = 34 participants to allow for potential dropouts. 3 subjects were excluded because more than 33 % of all epochs were excluded during pre-processing. One additional subject was excluded due to technical errors during the recording. This left *N* = 30 participants (25 female; mean age: 24.2 years, range: 19–35 years; 26 right-handed), all with normal or corrected-to-normal vision. Participants received course credit or €12/hour and provided written informed consent. All procedures adhered to the Declaration of Helsinki and were approved by the research ethics committee (Ethikbeirat) of the University of Leipzig (Approval No. 2025.04.07_eb_317).

### *2.2* Materials, stimuli, and task

Stimulation was controlled using custom scripts in Psychophysics Toolbox 3 (Brainard, 1997; Kleiner et al., 2007) implemented in MATLAB R2022b, running on Linux. Flicker stimulation was delivered via a PROPixx DLP LED projector (VPixx Technologies Inc., Canada) at a resolution of 960 × 540 pixels and a refresh rate of 480 Hz that projected on a screen 120 cm in front of participants.

**Fig. 1:**
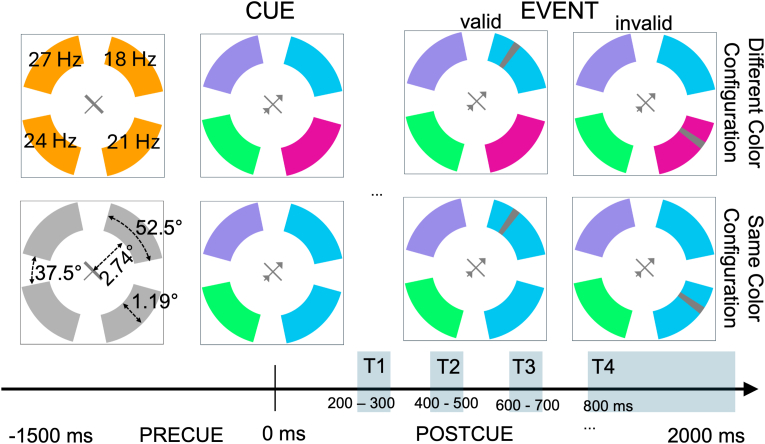
Trial scheme and stimulus events. Each trial consisted of a pre- and a post-cue time window. Time zero denotes the onset of the cue. Pre-cue: time before cue onset, −1500 (jitter: ±100 ms) – 0 ms. Post-cue: time after cue onset, 0–2000 ms. Each arc was colored identically during the pre-cue time window and presented with an on/off flicker throughout the trial with a fixed frequency (see upper left panel). At cue onset, a central arrow appeared, and arcs changed to one of two color configurations. Post-cue events, transient grey stripes, appeared either at the cued or an adjacent arc and had to be detected (speeded response). Events were equally likely to appear in one of the first three time bins (T1-T3). In one third of all event trials, the fourth time bin (T4) contained a second late event, which served to motivate subjects to maintain attention for a longer period at the cued location. See methods for details.

We presented four arcs and a central fixation as depicted in Fig. 1. During the pre-cue period, all arcs were identically colored, displayed on a dark grey background (23.5 cd/m^2^), and positioned equidistantly from a central x-shaped fixation cross (length of arms = 0.86° of visual angle [VA]) at an eccentricity of 2.74 VA and width of 1.19° VA. Each arc subtended 52.5° of a circle around fixation, followed by 37.5° of empty space. Throughout the trial, each arc flickered at a fixed frequency: upper right = 18 Hz, lower right = 21 Hz, lower left = 24 Hz, upper left = 27 Hz (see Fig. 1, upper left panel). The 3 Hz minimum frequency separation excluded frequency leakage of the applied Gabor Energy filter (Andersen and Müller, 2010; M. M. Müller et al., 2016). All flicker frequencies were synchronized to cue onset.

Five colors were defined, including one baseline color (counterbalanced across subjects) and four arc colors, with CIE-Lab coordinates at [59, 34.5, 59.75; orange], [59, −69, 0; cyan], [59, 69, 0; magenta], [59, 44.35, −52.86; purple], [59, −44.35, 52.86; lime]. Isoluminance was adjusted between the baseline color and all possible post-cue colors to avoid luminance-induced SSVEP amplitude changes between the pre- and post-cue period with heterochromatic flicker photometry (Wagner and Boynton, 1972). Each color was equally likely to occur at the cued location. At cue onset, arcs changed from the unique baseline color to either a different- (each arc had a unique color) or a same-color configuration (one of the adjacent arcs had the same color as the cued arc).

Targets were most likely at the cued location (different color configuration: *valid or same color configuration: valid match*, 33.34 % each) with uncued targets appearing either on a differently colored arc in the different-color configuration (*inval*, 11.1 %), a differently colored arc in the same-color configuration (*inval non-match*, 11.1 %), or on the same-colored uncued arc in the same-color configuration (*inval match*, 11.1 %).^1^ Color configurations were equally likely across trials (50 % of trials each). Events were equally likely across three 100 ms time bins (200–300 ms, 400–500 ms, 600–700 ms). Post-cue events were grey stripes (5° arc) with a transparency of 90 % of an opaque presentation, appearing at a random location within arcs. For all conditions, each location within the arc was equally likely. Post-cue targets required a space bar press as fast as possible (speeded detection task). Post-cue events were presented in 36 % of all trials (*N*_*post-cue-events*_ = 360). In one-third of all post-cue event trials (*N*_*late-events*_ = 120), a second late event appeared between 800 and 1850 ms, but not earlier than 800 ms after the first event onset. Late events appeared equally likely on the cued or an adjacent location and were not intended to be analyzed, but to motivate participants to maintain attention at the cued location until trial offset to allow for SSVEP amplitude time course analysis (see below).

To obtain a baseline SSVEP amplitude estimate without attention being deployed to one of the arcs, we presented pre-cue events that consisted of a transient thickening of one arm of the fixation cross (duration = 150 ms). Pre-cue events occurred in 16.8 % of trials (*N*_*pre-cue-events*_ = 168) during the pre-cue time only. Participants were instructed to press the space bar as fast as possible to report pre-cue events. With this pre-cue task we wanted to make sure that (a) attention is deployed to central fixation, and (b) that subjects shifted attention with the presentation of the cue.

### *2.3* Procedure

The fixation cross was visible on the screen throughout a block. Each trial began with the onset of the flickering arcs, all in baseline color. During the pre-cue period, participants were instructed to fixate and report events on the fixation cross. In every trial, after 1500 ms (±100 ms), a spatial cue indicated the to-be-attended arc. Participants were instructed to maintain central fixation while covertly shifting attention to the cued arc and pressing the space bar as quickly as possible when post-cue targets appeared (speed instruction). The subsequent trial began after an inter-trial interval of 800 ms.

Participants were informed about cue validity probabilities (“most events appear at the cued location”) and encouraged to focus on the cued arc for optimal performance. Blinking was encouraged during inter-trial intervals. After each block, feedback on the average response time compared to all previous participants was provided (e.g., “Your response time was x ms, that is y% faster/slower than average.”) to emphasize speeded responses.

The experiment took place in an electromagnetically and acoustically shielded chamber. Before EEG recordings, participants completed at least two training blocks of 25 trials each to familiarize themselves with the task and fixation. The full experiment consisted of 1000 trials in randomized order, with 360 trials containing post-cue events and 640 trials without post-cue events presented in 20 blocks. Each block lasted about 3.5 min and bloks were separated by breaks. Subjects started the next block whenever they felt ready. All in all the entire recording lasted between 80 and 90 min. Participants switched responding hand after 50 % of the blocks, with hand usage counterbalanced across subjects.

### *2.4* Behavioral data analysis

To test for task compliance, performance was assessed by extracting hit and false alarm rates for pre- and post-cue targets, averaged across cue conditions and color configurations, respectively. False alarms were defined as button presses in trials without events. Response times within ±3.5 standard deviations around the mean response time across all subjects and conditions, but not faster than 150 ms, were considered for analysis.

Response times were averaged per subject and condition (*val match*, *val*, *inval match*, *inval non-match* and *inval*) and subjected to a one-way repeated-measures ANOVA with the factor of Condition. The following planned contrasts were conducted (Holm-corrected): *val match vs. inval non-match*, and *val vs. inval* for spatial effects in response times and *val match vs. inval match* for a spatial effect within the same-colored elements, as well as *inval match vs. inval non-match* to test whether invalid events located on the same-colored uncued arc elicit faster responses than on the differently-colored arc.

To test whether the presence of a same-colored element in the display affected spatial selection, we compared spatial selectivity between color configurations. Therefore, we computed the difference in response time between *valid* and *invalid* events in both color configurations (*contrast same* = *inval non-match – val match*, *contrast diff = inval – val*). For each difference, a paired inferential, and, to secure evidence in favor of the null, a paired Bayesian *t*-test were conducted to compare these differences in response times. All Bayesian *t*-tests reported in this manuscript are implemented with a standard Cauchy Prior of *r* = 0.71.

The ANOVA was conducted using the *aov_car* function in the afex-package (Singmann et al., 2023), followed by pairwise comparisons between conditions via the emmeans-package (Lenth, 2023). Greenhouse-Geisser corrections were applied when necessary, and post-hoc comparisons were Holm-corrected. Effect sizes were computed using the *eff_size* function in the emmeans-package. Population standard deviation and degrees of freedom were based on the fitted linear model using the *lmer*-function of the lme4-package (Bates et al., 2015). Bayesian *t*-tests were conducted with the *ttestBF*-function from the BayesFactor-package (Morey and Rouder, 2022).

### *2.5* Analysis of electrophysiological data

#### *2.5.1* Data acquisition

EEG was recorded using 64 Ag/AgCl electrodes and two electrodes at the outer canthi of both eyes and vertically above and below the right eye were used to measure horizontal and vertical eye movements and blinks. Data were collected with an ActiveTwo Amplifier (BioSemi, The Netherlands) at a 512 Hz sampling rate with a 0.16 Hz high-pass and 100 Hz low-pass filter and stored for offline analysis.

#### *2.5.2* Pre-processing

Pre-processing was done using EEGLAB (Delorme and Makeig, 2004) and custom MATLAB scripts. Data were resampled to 256 Hz, and epoched from −1500 to 2200 ms relative to cue onset. Only trials without post-cue events were considered for epoching to avoid a modulation of SSVEP amplitudes by post-cue events attracting attention, especially by invalid events, and a possible contamination of SSVEP amplitude time courses by evoked potentials that were elicited by these events. Trials with pre-cue events remained in the data. Artefacts in the analysis window (−1000 to 2000 ms) were removed, including linear detrending, and discarding trials containing blinks (average number of trials discarded: *M* = 0.03 %; SD = 0.06 %) and eye movements (>25 μV, corresponding to approx. 1° of visual angle on the horizontal meridian; average number of trials discarded: *M* = 12.52 %; *SD* = 8.29 %). Additionally, epoched raw data was scanned visually to identify trials with remaining eye-movements or blinks. Then, noisy channels were identified using SCADS (Junghöfer et al., 2000) and spline-interpolated, discarding trials having more than 15 interpolated channels (mean proportion of rejected trials: *M* = 7.02 %; *SD* = 3.11 %; average number of channels interpolated: *M* = 3.44; *SD* = 0.94). Overall, an average of 19.71 % (*SD* = 8.07 %) of all trials without events were discarded and, on average, *M* = 513.9 trials per subject (*SD* = 51.6) entered the analysis. The remaining data were transformed to scalp current density (CSD) to pronounce local maxima of neural signals (Ferree, 2006; Kayser and Tenke, 2015).

#### *2.5.3* SSVEP analysis

SSVEP analysis focused on stimulus-evoked amplitudes in the analysis window (−1000 to 2000 ms). Pre-processed trials were averaged per condition: *cued match* = cued arc in same color configuration, *cued* = cued arc in different color configuration, *uncued match* = uncued arc in same color as cued arc, *uncued non-match* = uncued arc colored differently than the cued arc and originating from the same color configuration, and *uncued* = uncued arc in the different color configuration. The arc located diagonally to the cued arc was not included in the analysis. The uncued conditions were pooled across trials with the uncued position located either clockwise or counterclockwise to the cued arc. For example, *uncued match* at lower right position could originate either from a trial cueing the upper right or the lower left arc, with the same-colored arc located in the lower right position. To ensure equal signal-to-noise ratio for each condition, a random selection of 50 % of the trials for the *uncued* condition in the different color configuration was selected for trial averaging (see Fig. S1 in the Supplementals for illustration of trial averaging).

Then, data were detrended and Fourier-transformed (for time window of 3 s, with a sample rate of 256 Hz, resulting in a frequency resolution of 1/3 Hz). The absolute values of the Fourier coefficients were extracted for each subject, channel, and condition and averaged across the analysis window (from −1000 to 2000 ms relative to cue onset) within each subject to test for the signal-to-noise ratio of SSVEP amplitudes at the stimulated frequencies. Electrode selection was performed for each subject and frequency individually, based on the electrode showing the maximum SSVEP amplitude within a predefined parietal-occipital cluster (I1, I2, Iz, O1, O2, Oz, P10, P5, P6, P7, P8, P9, PO3, PO4, PO7, PO8, POz; see Fig. 3). Selecting the individual best electrodes across the analysis window including pre- and post-cue time (from −1000 to 2000 ms) can maximize the electrode selection to detect an SSVEP amplitude enhancement but may obscure suppressive effects. However, we chose a broader analysis time window for electrode selection for the following reasons: (1) Cued locations varied trial-by-trial with equal probability across frequencies ensuring that enhancement and putative suppression effects were evenly distributed across frequencies. As a result, the influence of the spatial cue on amplitude enhancement should affect electrode selection similarly across all frequencies. (2) If electrode selection had been based on the pre-cue time only, the change of color between pre- and post-cue would introduce a potential selection bias for the contrast of the baseline color (and its contrast to the background, cf. Norcia et al., 2015). Furthermore, with the individual best electrode selection, we account for variations in topographies for the SSVEP amplitudes between subjects and frequencies (see Fig. S3 in the Supplementals).

The electrode with the maximum amplitude was complemented by four neighboring electrodes, resulting in five electrodes per subject and frequency. This electrode selection combines the benefits of choosing the electrode with maximal amplitude with the smoothing of time courses when averaging across a cluster of electrodes. SSVEP quality was assessed by comparing the amplitudes at the flicker stimulation frequencies with sideband amplitudes (±1 Hz of the respective stimulation frequency) within the analysis window (−1000 to 2000 ms). A two-by-four repeated-measures ANOVA was conducted with the factors of Band (peak vs. side) and Frequency (18, 21, 24, 27 Hz).

##### *2.5.3.1* Analysis of Gabor filtered time courses

Pre-processed trials without post-cue events were averaged per subject, cue position, and channel. Then, time courses of SSVEP amplitudes were analyzed using a Gabor-energy filter centered at each frequency of stimulation (FWHM_frequency_ = ± 1 Hz; FWHM_time_ = ± 220.636 ms). The time courses of SSVEP amplitudes were extracted for each subject, frequency, condition, and channel. After filtering, data were averaged across selected channels for each frequency and baseline-corrected (−720 to −220 ms). This baseline not including zero was chosen to account for the temporal resolution of the Gabor filter to ensure that no post-cue data points would contribute to the baseline values. Pre-to post-cue SSVEP amplitude modulation was calculated as the percentage of change in post-compared to pre-cue amplitude and then collapsed across frequencies (Cohen, 2014, see also Gundlach et al., 2023a, Gundlach et al., 2023b for a similar approach).

Statistical analysis used cluster-corrected running *t*-tests (one-tailed) to compare time courses of SSVEP amplitudes of the same color configuration (*cued match*, *uncued match*, *uncued non-match*) and the different color configuration (*cued, uncued*) against baseline. To test neural temporal dynamics of stimulus enhancement in the same color configuration, running *t*-tests contrasted the time course of the cued arc with the uncued same-colored arc (*cued match vs. uncued match*) and the uncued differently-colored arc (*cued match vs. uncued non-match*). The global feature spread was tested by the contrast between the uncued same-colored and the uncued differently-colored arc (*uncued match vs. uncued non-match*). To test stimulus enhancement in the different color configuration, running *t*-tests contrasted the time course of SSVEP amplitudes for the cued arc with the uncued differently-colored arc (*cued match > uncued match*). The time window of analysis for all running *t*-tests was set from 0 to 1500 ms. Cluster-correction involved comparing summed *t*-values of clusters with *p*-values >0.05 to the distribution of repeatedly calculated summed *t*-values for substitute data (5000 iterations) derived from shuffled condition labels (Maris and Oostenveld, 2007). Shuffling the condition labels corresponds to the assumption under the null hypothesis of no difference between pre- and post-cue SSVEP amplitudes (in case of tests against zero) or no differences between conditions (Cohen, 2014).

Since our core interest to investigate the time course of SSVEP amplitude enhancement for the cued and uncued, but same-colored arc included the assumption of a simultaneous amplitude enhancement and, thus, a Null effect as a possible outcome, we implemented a Bayesian analysis of time courses, as in previous work (de Lissa et al., 2020). For this goal, we contrasted SSVEP amplitude modulation of the cued and the uncued, same colored arc, time point by time point with a Bayesian *t*-test. We expected that the Bayes Factors for this contrast should be lower than 0.33 to accept the Null hypothesis that the two time courses show no differences in their trajectory (Lee and Wagenmakers, 2014). The time window of analysis for these running Bayesian *t*-tests was identical to the running *t-*tests.

To assess whether the presence of same-colored arcs influenced the temporal dynamics of SSVEP amplitudes, we compared the onset times of SSVEP amplitude enhancement of the *uncued match* and *uncued* conditions (different color configuration). Therefore, we implemented a jackknife approach to retrieve a distribution of individual onset times for each condition (cf. Gundlach et al., 2020). We retrieved 30 subsamples by excluding each subject label once, resulting in a subsample size of N-1. The average SSVEP amplitude time course was computed for each subsample and condition. Onset times were defined as the time point at which the amplitude reached 85 % of the base-to-peak SSVEP amplitude range, based on each subsample's time course. As onset time extraction can be considerably biased by noise extraction (Kiesel et al., 2008; Liesefeld, 2018), we chose this threshold instead of the 50 % often employed for ERPs. This 50 % criterion would correspond to a SSVEP amplitude lower than the baseline-enhanced response observed in either condition, thereby increasing the risk of capturing noise rather than signal. Therefore, we chose a criterion that clearly corresponds to SSVEP amplitude values higher than the amplitude at which the grand mean average time course of each condition deviates from baseline significantly, which was the case for the 85 % criterion. Individual onset times were obtained from these subsample onset times according to Smulders (2010) and subjected to a paired *t*-test (two-tailed).

##### *2.5.3.2* Analysis of averaged post-cue SSVEP amplitudes

Three additional contrasts were computed to assess the effect of color configurations on the baseline-corrected SSVEP amplitudes, averaged over a time window of 750 ms–1250 ms. First, we were interested in whether the SSVEP amplitude enhancement for the cued arc, i.e., spatial facilitation, differed between color configurations. Therefore, we contrasted the averaged SSVEP amplitudes for the cued arc between the same and the different color configuration using a paired *t-*test. In the case of a non-significant result, we assessed the probability of the Null hypothesis given the data (i.e., no difference between conditions) by a Bayesian *t*-test. Second, we contrasted spatial selectivity, defined as the difference between the SSVEP amplitude for the cued and the uncued different-colored object, between color configurations. Therefore, we computed the difference between *cued* and *uncued* (different color configuration) and the difference between *cued match* and *uncued non-match* for each subject and contrasted these differences by a paired *t*-test and applied a Bayesian *t*-test in case of a non-significant result. Third, we contrasted the SSVEP amplitudes for the uncued same-colored arc with uncued different-colored arc in the different color configuration to assess the feature effect across color configurations by a paired *t*-tests and Bayesian *t*-test.

## *3* Results

### *3.1* Behavioral results

Analysis of pre-cue task performance revealed very high hit rates (*M* = 97 %, *SD* = 4.7 %; False-Alarm-Rate: *M* = 0.1 %, *SD* = 0.2 %). Post-cue task performance was high as well (Hit rate: *M* = 81.8 %, *SD* = 8.8 %; False alarm rate: *M* = 1.5 %, *SD* = 1.5 %), clearly showing that participants were compliant to instructions and task.

**Fig. 2:**
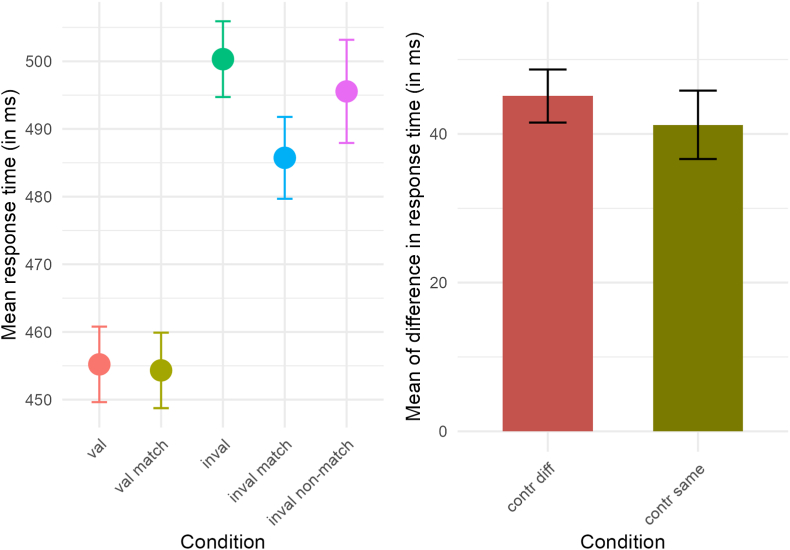
Mean response time in each condition for the post-cue task. Left panel: „val” = target appears on the cued arc and all elements are colored differently, “val match” = target appears on cued arc and one other (adjacent) element is colored identical, “inval” = target appears on the uncued differently colored arc and all arcs are colored differently, “inval match” = target appears on the uncued arc of the same color as the cued arc, “inval non-match” = target appears on the uncued differently colored arc and two arcs in the display are colored identically. Right panel: „contr same” = mean of difference in response time between validly and invalidly cued events on the different colored arc, when two arcs are colored identically, “contr diff” = mean of difference in response time between validly and invalidly cued events on the different colored arc, when all arcs are colored differently. Error bars represent SEM.

Average response times for each condition are depicted in Fig. 2. The ANOVA of response times revealed a main effect of Condition, *F*(2.74, 79.39) = 61.46, *p* < 0.001, *η*^*2*^ = 0.26. In the same color configuration, responses to events on validly cued arcs were 40.34 ms faster than to invalidly cued events on the differently colored object, *t*(29) = −8.44, *p* < 0.001, *d* = −2.62. Responses to valid events were 31.41 ms faster than to invalid events on the same colored arc, *t*(29) = −7.41, *p* < 0.001, *d* = −2.04. Responses to invalid events on the same colored arc were 9.81 ms faster than responses to invalid events on the differently colored arc, *t*(29) = −2.12, *p* = 0.043, *d* = −0.64.

**Fig. 3:**
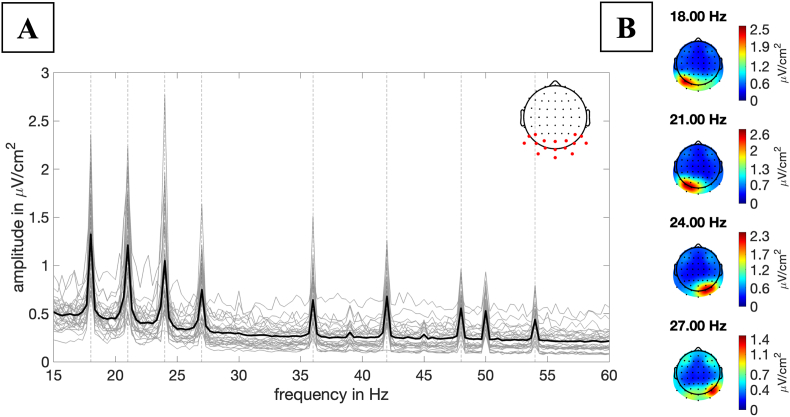
FFT Results. **(A)** Grand mean spectra for all subjects (N = 30) across all conditions for the combined electrode cluster of left and right hemifield stimulation in the analysis time window (−1000 to 2000 ms relative to cue onset). Grey lines indicate single-subject spectra. Vertical lines indicate frequencies of stimulation and their respective harmonics. Red channels indicate the parietal-occipital electrode cluster predefined to determine the individual best electrode (I1, I2, Iz, O1, O2, Oz, P10, P5, P6, P7, P8, P9, PO3, PO4, PO7, PO8, POz). **(B)** Grand Mean topographies for each frequency in the same time window. One frequency was fixed to one arc: upper right: 18 Hz, lower right: 21 Hz, lower left: 24 Hz, upper left: 27 Hz (cf. Fig. 1). Note: Different scales for the topographies of each frequency.

In the different color configuration, responses to events on validly cued arcs were 45.1 ms faster than on invalidly cued arcs, *t*(29) = −12.62, *p* < 0.001, *d* = −2.93. Spatial selectivity in both color configurations, represented by the difference between valid and invalid different events (*inval* - *val*, *inval non-match* - *val match*) revealed no difference between color configurations, *M*_*diff*_ = 3.88 ms, *t*(29) = 0.93, *p* = 0.361, *d* = 0.17, supported by evidence in favor of the null, *BF*_*10*_ = 0.20 ± 0.00034, indicating that the null is 5 times more likely given the data.

### *3.2* Electrophysiological results

#### *3.2.*1 FFT-transformed data

The Grand Mean Spectra and topographies for the time window -1000–2000 ms relative to the cue are depicted in Fig. 3. The Grand Mean topographies showed the expected lateralization following the stimulus location relative to central fixation (right: 18 and 21 Hz, left: 24 and 27 Hz). The analysis of amplitudes of peak-vs sideband per frequency in the same time window revealed a main effect of Frequency, *F*(2.48, 72.02) = 18.74, *p* < 0.001, *η*^*2*^ = 0.125, and a main effect of Band (side vs. peak), *F*(1, 29) = 105.01, *p* < 0.001, *η*^*2*^ = 0.337 as well as an interaction, *F*(2.62, 75.96) = 13.37, *p* < 0.001, *η*^*2*^ = 0.059. Pairwise comparisons between levels of Band indicated substantial signal-to-noise ratio for all frequencies (see Table A1 in the Supplementals).

#### *3.2.2* Time courses of SSVEP amplitudes

##### 3.2.2.1 Same color configuration

**Fig. 4:**
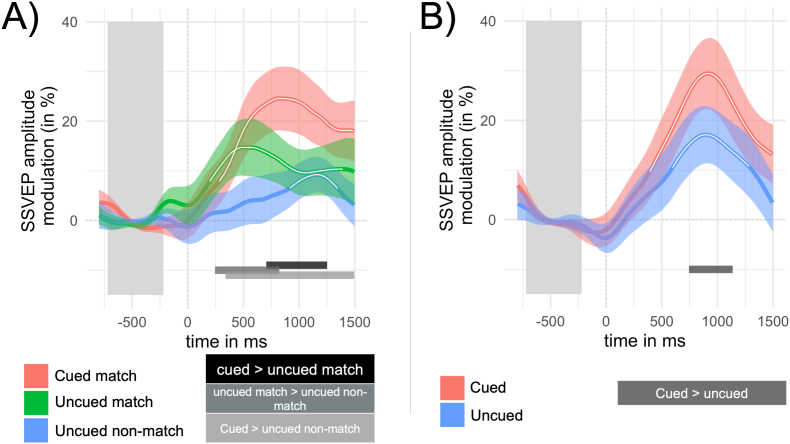
SSVEP Amplitude Time Courses. **(A)** SSVEP amplitude time courses for same color configuration. “Cued match” = cued arc when one adjacent arc was colored identically, “uncued match” = uncued arc is colored identically to the cued arc, “uncued non-match” = uncued arc is colored differently compared to the cued arc. **(B)** SSVEP amplitude time courses for different color configuration. “Cued” = cued arc, all arcs are colored differently, “uncued” = uncued arc. Colored lines are SSVEP amplitude time courses of each condition. Error patches represent SEM. White lines indicate significant cluster-corrected time points of each condition against pre-cue baseline. Greyscale bars below indicate significant time points for respective contrasts. The darker grey area represents the baseline period (−720 to −220 ms). Time zero corresponds to the onset of the spatial cue.

The time courses of SSVEP amplitude modulations in the same color configuration are depicted in Fig. 4A. As indicated by the cluster-corrected permutation tests against pre-cue baseline, the SSVEP amplitudes for the cued arc in the same color configuration started to be significantly different from baseline (pre-cue window) 283 ms after cue onset until the end of the analysis window. For SSVEP amplitudes elicited by the uncued same-colored arc in the same color configuration this was the case between 197 and 1393 ms after the cue. SSVEP amplitudes for the uncued differently colored arc exhibited a much later time point when this significant change occurred, between 916 and 1345 ms, relative to baseline.

The contrast between SSVEP amplitudes for the cued and uncued same-colored arc resulted in a significant cluster from 705 to 1252 ms, indicating higher SSVEP amplitudes for the cued arc in this time window. The contrast between the uncued same-colored arc and the uncued differently-colored arc revealed a considerably earlier cluster (244–822 ms). The contrast between the cued and the uncued differently-colored arc was significant at about 338 ms until 1494 ms.

The time course of Bayes Factors for the Bayesian *t*-tests between SSVEP amplitudes elicited by the cued and the uncued same-colored arcs remained below 0.33 until approximately 588 ms relative to the cue, indicating that SSVEP amplitude time courses were in favor of the Null for these time points. Note that this time point is (on a descriptive level) later than the time point when both conditions differed from baseline, indicating a significant amplitude enhancement effect for the cued and the uncued same-colored arc.

##### 3.2.2.2 Different color configuration

The time courses of SSVEP amplitude modulations in the different color configuration are depicted in Fig. 4B. SSVEP amplitudes for the cued arc in the different color configurations were enhanced relative to pre-cue baseline at about 385 ms after cue onset until the end of the analysis window. SSVEP amplitudes for the uncued arc in this configuration were enhanced considerably later between 588 and 1291 ms after the cue. The contrast between SSVEP amplitudes for the cued and the uncued arc revealed significant differences between 744 and 1174 ms, indicating higher SSVEP amplitudes for the cued arc than the uncued arc.

##### 3.2.2.3 Statistical analysis of onset times between SSVEP amplitude time courses

The distribution of onset times of SSVEP amplitude time courses for *uncued match* and *uncued* is depicted in Fig. S5 in the Supplementals. Average onset times for the SSVEP amplitudes of *uncued match* (*M*_*uncued-match*_ = 351 ms*; SD*_*uncued-match*_ = 458 ms) occurred earlier than average latencies for *uncued* (*M*_*uncued*_ = 725 ms*; SD*_*uncued*_ = 480 ms), *t*(29) = −3.14, *p =* 0.0038, *d* = −0.574. This clearly shows that facilitation of the uncued, same color arc occurred significantly earlier than facilitation of the uncued arc when no matching colors were present.

#### 3.2.3 Comparison of Sustained effects between color configurations

Facilitation of the respective cued arc as indicated by the modulation of SSVEP amplitudes compared to baseline (averaged across 750 ms–1250 ms), did not differ between color configurations, (*cued* vs *cued match*), *M*_*diff*_ = −3.01 %, *t*(29) = −0.46, *p* = 0.677, *d* = −0.08, supported by evidence in favor of the null, *BF*_*10*_ = 0.22 ± 0.00034. In line with spatial facilitation, spatial selectivity as indicated by the difference between amplitudes for the cued and the uncued differently colored arc was equivalent between stimulus configurations, *M*_*diff*_ = −4.70 %, *t*(29) = −0.633, *p* = 0.734, *d* = −0.20, supported by evidence in favor of the null, *BF*_*10*_ = 0.23 ± 0.00033. Surprisingly, there was no difference in mean SSVEP amplitudes between the uncued same-colored arc and the uncued different-colored arc originating from the different color configuration in that analyzed time window (*uncued match* vs. *uncued*), *M*_*diff*_ = −4.62 %, *t*(29) = −1.06, *p* = 0.851, *d* = −0.19, supported by evidence in favor of the null, *BF*_*10*_ = 0.32 ± 0.00032.

## *4* Discussion

In a probabilistic spatial cueing experiment in which we cued one out of four arcs, we aimed to investigate whether the cued arc/object would result in strictly object-based processing that restricts facilitation of features to the cued object, or whether we can observe global feature-based spread across object boundaries, resulting in facilitation of an uncued arc/object that shares the same color as the cued one. A second aim was to uncover temporal dynamics when such global feature-based spread occurs. As outlined in “Introduction”, two contradictory results were recently reported. On the one hand, time delayed (Andersen and Hillyard, 2024), on the other hand, temporally parallel global feature-based spread (Gundlach et al., 2023b). Employing frequency-tagging as a perfect and powerful tool to investigate such neural temporal dynamics in early visual cortex (Boylan et al., 2023; Di Russo et al., 2007; M. M. Müller et al., 1998b), we found feature-based global spread that was not confined to object boundaries. This spread occurred in a temporally parallel fashion, once attention was shifted to the cued arc, replicating the results of our previous study (Gundlach et al., 2023b).

Results are in line with behavioral (Chapman and Störmer, 2021; Gonen et al., 2014) and neurophysiological evidence (Adamian et al., 2020; Boehler et al., 2011) demonstrating global feature-based spread across object boundaries. Behavioral data of the present study were also supportive of that notion: Response times to invalid events were faster when they appeared on a same-colored compared to a differently colored arc. Since the distance between the cued and the uncued arc was identical for the same and differently colored arcs, and target events were distributed equally across the spatial extent of these arcs, we can exclude a spatial explanation for this result and attribute this effect to global feature-based facilitation.

In line with behavioral data, neural responses clearly support global feature-based spread by (a) a significant enhancement of SSVEP amplitudes (compared to baseline) for the uncued arc matching the color of the cued arc and (b) higher SSVEP amplitudes elicited by the uncued arc matching the color of the cued arc compared to the uncued arc not matching the cued arc (both originating from the same-color configuration). These differences in SSVEP amplitudes fit well with the idea of global feature-based sensory gain of neurons that code the respective (target) color (Andersen et al., 2008, 2011; Forschack et al., 2017; Gundlach et al., 2022; Gundlach et al., 2023a, Gundlach et al., 2023b; M. M. Müller et al., 2018; Treue and Martínez-Trujillo, 1999). Interestingly, the time point of this feature effect is at about the time window of the selection negativity (SN), an event-related potential (ERP) component that was linked to feature-based processing with a latency of about 180–250 ms on average (Anllo-Vento and Hillyard, 1996; Hillyard and Anllo-Vento, 1998). This coincides nicely with the first time points of a significant SSVEP amplitude increase relative to baseline of the present study, suggesting neural activation patterns in feature-specific cortical areas with such a latency. In fact, in a recent combined functional magnetic resonance (fMRI) and EEG-SSVEP study, we have shown that when subjects attended to colored compared to greyscale stimuli, attentional SSVEP amplitude enhancements were mainly driven through sensory gain in area V4 (Boylan et al., 2023).

Our global effect of feature-based spread across object boundaries, as measured with SSVEP amplitudes, is in line with previous work (Adamian et al., 2020; Boehler et al., 2011). Boehler and colleagues presented two circular spheres in each hemifield. Each sphere was composed of two differently colored parts with orthogonal convexity. Participants’ task was to report the convexity of a red half (left or right) by pressing a button. The other color of the target sphere (e.g., green) was not task-relevant. However, this color (green in the example) either matched the color of a second sphere located in the opposite hemifield or did not match. Boehler and colleagues reported an ERP modulation with a latency of about 80 ms relative to the target evoking N2pc (reflects target processing) for the element at the opposite side with a color match, suggestive of global feature-based spread across object boundaries. As outlined in “Introduction”, Adamian and colleagues (2020) also reported global enhancement for the attended feature across the visual field, measuring SSVEP amplitudes. Different from the present study and the one from Boehler and colleagues, this study used two spatially fully overlapping RDKs that differed in rotation direction to form two semitransparent surfaces. Therefore, it cannot be excluded that the observed feature spread across the two surfaces was a consequence of this spatial superimposition.

However, identical to one of our previous studies (Gundlach et al., 2023a, Gundlach et al., 2023b), all these studies used a color or greyscale cue to guide subjects’ attention to a certain color/greyscale of a certain object. This color cue allows for the build-up of a color template, stored in working memory, that might be responsible for global feature (color) based spread. Contrary to these studies, the present study merely provided a cue indicating a specific location (arc). This cue lacked color information, precluding the construction and storage of a corresponding color template. Nevertheless, we found global feature-based spread that adds an important aspect in feature-based processing, suggesting automatic feature facilitation even without a color template in working memory, i.e., essentially stimulus-driven. Note, arcs turned into their specific colors with the onset of the spatial cue.

Analysis of neural temporal dynamics revealed that facilitation of the cued and same-colored arc occurred in parallel, with no time delay between the facilitation of the cued and the same-colored arc. Although the first significant time points of cluster-corrected permutation tests compared to baseline were not numerically identical for both arcs, Bayesian analysis of the SSVEP amplitude differences between these two arcs showed evidence that they did not differ during the first 580 ms after the cue. In other words, global feature-based spread occurred *parallel* to the selection of the cued location*.* This finding supports the notion that feature-based processing was ignited when the attentional spotlight (Posner, 1980) was shifted to the cued arc. This underlines the automatic and obligatory nature of global feature-based processing in early visual cortex (Andersen et al., 2013).

Automatic and parallel feature (color) processing was also demonstrated by the fact that SSVEP amplitudes (compared to baseline) elicited by the uncued arc matching in color appeared significantly earlier than for the uncued arc originating from the different color configuration (see Fig. S5 in Supplementals) and for the uncued arc with a different color in the same color configuration (see Fig. 4A). The finding that these uncued arcs in both configurations were also facilitated can easily be explained by the fact that subjects were aware and learned that invalid targets occurred in these arcs as well, what most certainly resulted in a broadening of the attentional spotlight, including the adjacent arcs, but just as a secondary process with a significant time delay. This supposedly broadening of the attentional spotlight most certainly resulted in the same SSVEP amplitudes at the later time window between the uncued arc in the different color condition and the same-colored arc in the same color condition, but, again, this happened at a significantly later time point that both had about the same amplitude magnitude (see Fig. S5 in Supplementals), again demonstrating that this broadening of the attentional spotlight is a secondary process. It should also be noted that subjects most certainly learned that after 700 ms only very few targets were presented (only 12 % of all targets in the experiment). Therefore, it is very likely that attentional resources were withdrawn from the stimuli after a second or so, what explains the reductions in SSVEP amplitudes for all stimuli after about a second after the cue (see Fig. 4).

Importantly, results clearly demonstrated that more attentional resources were deployed to the cued arc for the entire stimulation period, regardless of the stimulus configuration, with significantly greater SSVEP amplitudes compared to all other arcs. Such greater deployment of attentional resources to the cued arc was also supported by behavioral responses with significantly faster RTs to targets at the cued arc. The comparison of SSVEP amplitudes in the later window showed that they were identical for the cued arc in both stimulus configurations. In other words, global feature-based spread did not produce costs for the cued arc, neither in behavior nor in SSVEP amplitudes. In addition, the shifting time of attention to the cued location was basically identical for both stimulus configurations, a result paralleled in behavior (see Fig. S2 in Supplementals).

The time course of behavioral data also demonstrated that subjects focused attention on central fixation during the pre-cue period and shifted attention to the cued arc with cue onset. As depicted in Fig. S2 (Supplementals), both time courses of RTs clearly showed speeding up of RTs in the early time window between 200 and 300 ms. Such a speed-up in RTs before the level of best performance is reached is a typical pattern in spatial shifting, and the observed time before best performance was reached fits very well with latencies reported for the attentional dwell time (Ward et al., 1996) or shifting of the attentional spotlight after an instructive cue (Müller and Rabbitt, 1989).

As with “absolute” SSVEP amplitude for the cued arcs, temporal dynamics of location-based selection were unaffected by the presence of a same-colored element in the display. Costs were only observed for the uncued arc in the same color configuration that had a color different from the cued one, with the lowest SSVEP amplitudes for all arcs. Interestingly, despite the lowest SSVEP amplitude, we observed no consequences in behavioral responses. RTs to targets were about the same as for an uncued arc in the different color condition. Nevertheless, to us, these results are evidence that feature-based facilitation was triggered by the cued arc, commencing when attention was deployed to that location, despite the fact that global color facilitation and even color processing per se were not required to perform the task.

One interesting prediction of the feature-similarity gain model (Martinez-Trujillo and Treue, 2004; Maunsell and Treue, 2006) was that location does not hold any unique status; it represents merely another feature of the stimulus. This prediction cannot be held in this strong version. In line with the present study, previous studies in which spatial processing was combined with feature processing (Adamian et al., 2019; Andersen et al., 2011) demonstrated that neural responses to spatial processing/selection were always greater compared to responses to feature processing, indication that spatial processing is different from feature processing (but see Gundlach et al., 2023b, who were unable to replicate this location effect). It is noteworthy that we recently reported spatial prioritization (Shomstein and Yantis, 2002, 2004), i.e., significantly greater SSVEP amplitudes for an attended location of an object in object-based processing as well (Wehle et al., 2025), again suggesting a special role for spatial/location processing in integrated object processing (O'Craven et al., 1999).

What might have caused the difference in temporal dynamics of global feature-based spread between the study by Andersen and Hillyard (2024) and our study (Gundlach et al., 2023b), despite using very similar designs? We speculated that presenting RDKs with the relevant colors in the pre-cue period already might have caused pre-activation of color-sensitive neurons that code the two colors (red and blue in that case), resulting in temporally parallel global feature-based spread across the to-be-attended and to-be-unattended side of the screen. However, identical to the study by Andersen and Hillyard (2024), in the present study, all stimuli started to flicker in a different color from the final display, and we replicated parallel global feature-based spread, and as mentioned above, without the possibility to build up a color template. Interestingly, in the study by Andersen and Hillyard, subjects were instructed to which color and side they needed to attend to 800 ms before the color change, enough time to build up a color template and shift attention to the cued side. The present and our previous study (Gundlach et al., 2023b) clearly point to the direction that shifting attention in space was the time-consuming process. Introducing a symbolic spatial cue resulted in a significantly prolonged spatial shifting time compared to a simple arrow cue. Temporally parallel global feature-based spread occurred with both types of spatial cues. In other words, spatial selection is slow, as had already been concluded by Ward and colleagues (1996), whereas feature selection is fast. To what extent spatial shifting processes might have caused the delay in the study by Andersen and Hillyard (2024) should be addressed in future research, but behavioral results suggest that spatial shifting was not completed 800 ms after spatial cue onset. Hit rates as well as RTs reached their level of best performance about 400 ms after RDKs changed to colors (see Fig. 2 in Andersen and Hillyard, 2024), which is a remarkably long latency, given that 800 ms should have been enough time to shift attention to the cued side.

One limitation of our study is a confound between spatial and object-based processing. The increase in the SSVEP amplitude of the cued arc compared to baseline can also be attributed to a purely spatial mechanism. Due to the spatial overlap of the observed space with the object presentation and cue position at the center of the object, spatial and object-based processing mechanisms make the same predictions. However, we base our work on the stimulus material from Gonen and colleagues (2014), who have already demonstrated feature- and object-based processing mechanisms for these arcs.

In summary, we showed global feature-based spread to same-colored elements across object boundaries in a probabilistic spatial attention task. Once spatial attention was shifted to the cued location, SSVEP amplitudes elicited by the cued arc and the same-colored uncued adjacent arc increased in a temporally parallel manner. Color processing was not required to perform the task, indicating, again, that global feature-based spread ocurred automically, independent of task demands. SSVEP amplitudes as well as behavioral responses nevertheless demonstrated that more attentional resources were deployed to the cued task throughout the presentation period. Interestingly, the presence of a same-colored uncued arc was not linked to costs for the cued arc, neither in SSVEP amplitudes nor in behavior.

## CRediT authorship contribution statement

**S. Wehle:** Writing – review & editing, Writing – original draft, Visualization, Software, Methodology, Investigation, Formal analysis, Data curation, Conceptualization. **C. Gundlach:** Methodology, Formal analysis, Conceptualization. **M.M. Müller:** Writing – review & editing, Supervision, Resources, Project administration, Funding acquisition, Conceptualization.

## Funding information

The research was funded by the 10.13039/501100001659German Research Foundation (10.13039/501100001659Deutsche Forschungsgemeinschaft), fund to M.M.M. (MU 972/27-1) and by the Open Access Publishing Fund of Leipzig University supported by the German Research Foundation within the program Open Access Publication Funding.

## Declaration of competing interest

The authors declare that they have no known competing financial interests or personal relationships that could have appeared to influence the work reported in this paper.

## Data Availability

Data will be made available on request.

## Footnotes

1 We intended to implement a few catch trials in which we present targets at the arc opposite to the cued arc. Unfortunately, these targets were not delivered due to a coding error. We do not think that this coding error had an influence on the overall results, given that we never intended to analyze behavioral responses or SSVEPs for that arc to keep the distance between the cued and the uncued arc(s) constant for all comparisons.
