# Supplementary material for: Temporally parallel facilitation of same-colored objects beyond spatial selection
Source: Neuroimage Rep. 2025 Nov 26;5(4):100302. doi: 10.1016/j.ynirp.2025.100302 (PMC12689214; doi:10.1016/j.ynirp.2025.100302)
Supplement: Multimedia component 1 [file mmc1.docx]

Supplementals to “Temporally parallel facilitation of same-colored objects beyond spatial selection”

## S1. Illustration of Trial Averaging


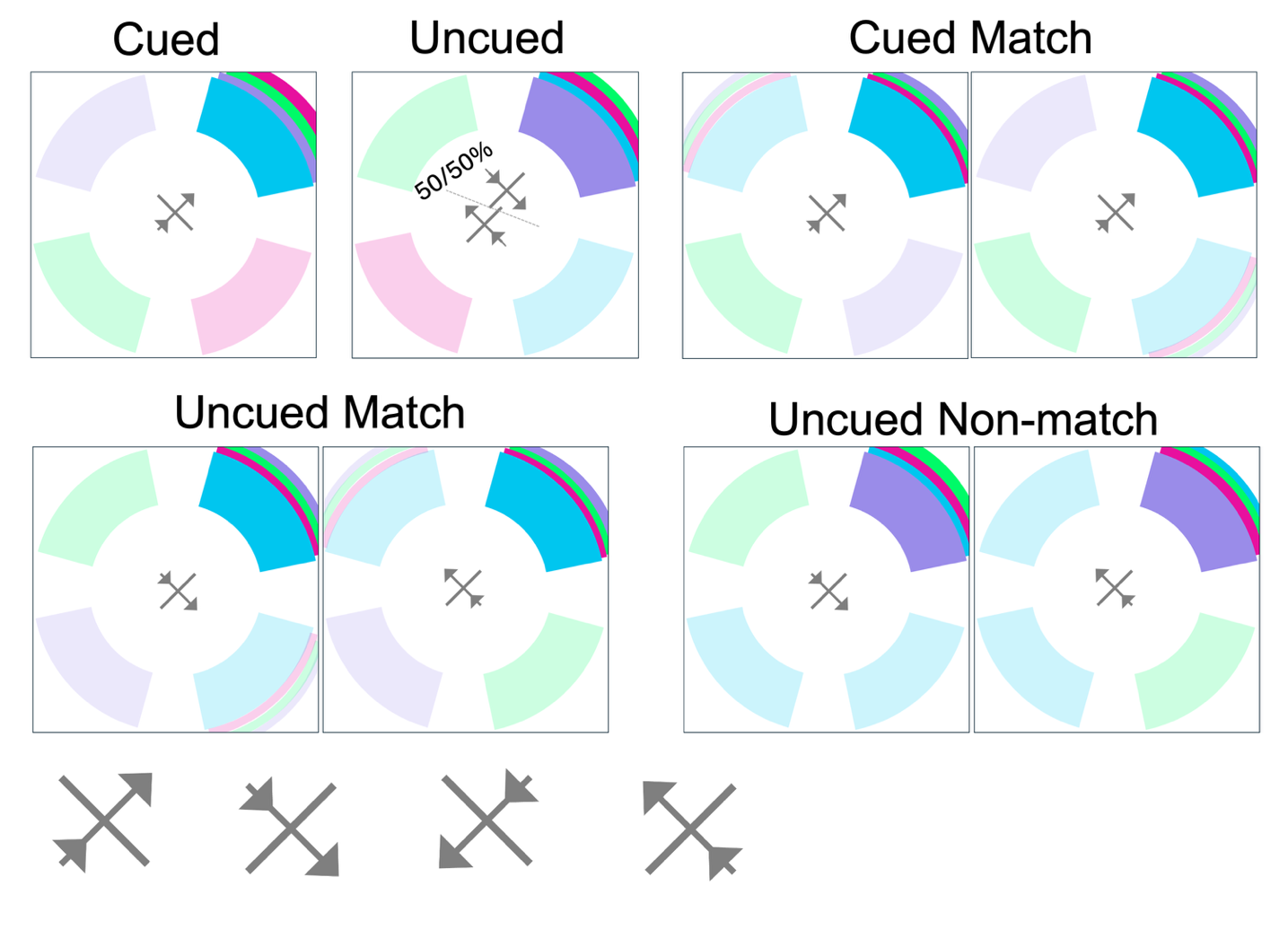


Figure S1: Trial Averaging. Illustration of trial averaging before applying the transformation from time into the frequency domain to obtain SSVEP amplitudes and time courses. Averaging is illustrated for the upper right arc that flickered at 18 Hz. The same logic was applied to all four cue/arc positions. The arc of interest is depicted as opaque, while the other arcs are shown as transparent. To ensure equal SNR across conditions, for the uncued arcs trials originated from a random selection of 50% of suitable trials. For example, for the object at the upper right location, suitable trials for this condition contained cues to upper left and lower right locations.

The logic of trial-averaging is shown in Figure S1. In the design, the uncued, same-colored object could be located clockwise or counterclockwise relative to the cued arc with equal probability. Consequently, the number of trials of each (nuisance) type in the same-color configuration was 50% compared to the number of trials in the different-color configuration (N_same-color-clockwise_ + N_same-color-counterclockwise_ = N_different-color-configuration_), to allow equal signal-to-noise ratio for both color configurations. As a result, twice as many trials were possible in the *uncued* condition (different color configuration) because uncued different-colored arcs were present clockwise and counterclockwise adjacent to the cued arc, unlike in the same-color configuration where only one uncued different-colored arc was present adjacent to the cued arc (N_different-color-clockwise_ + N_different-color-counterclockwise_ = 2*N_same-color-configuration_). Therefore, 50% of trials were randomly chosen for the *uncued* condition*.*

## S2. Time Course of Response Times


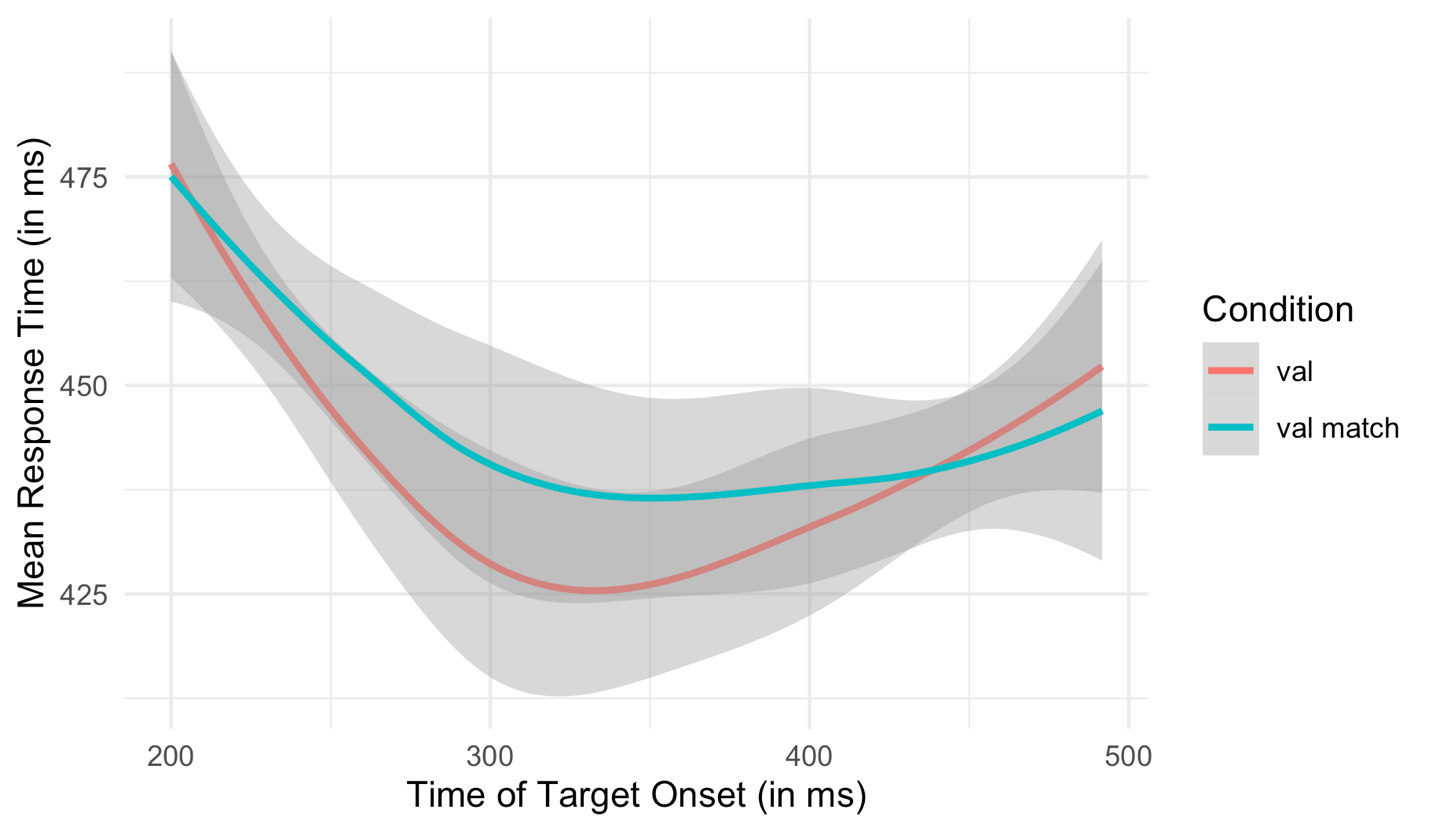


Figure S2: Time Course of Response Times for Valid Events. “Val match” = response times to valid events in the same color configuration. “Val” = response times to valid events in the different color configuration. Time of Target Onset indicates the onset time at which the post-cue target event appeared. LOESS smoothing (Locally Estimated Scatterplot Smoothing) was applied to highlight the temporal deployment in response times. The shaded regions represent the 95% confidence intervals for each condition’s smoothed curve.

Time courses of response times (RTs) to validly cued events are depicted in Figure S2. To visualize the time course of RTs as a function of post-cue target onset time relative to cue onset at time point zero (Time of Target Onset), we first aggregated all trials with valid events (*val match, val)* at the subject level before computing group-level averages. Response times within ± 3.5 standard deviations around the mean response time across all subjects and conditions, but not faster than 150 ms, were considered for analysis. Each subject’s mean RT was computed separately for each unique combination of the Time of Target Onset and Condition and was then averaged across subjects. To derive a continuous time course of RTs, we applied local polynomial regression fitting (LOESS) using the geom_smooth(method = “loess”) function in ggplot2 (Wickham, 2016) with a default LOESS span of 0.75, meaning that each local regression incorporated approximately 75% of the data to estimate the reaction time at each time point. Both time course of RTs clearly showed speeding up of RTs in the early time window between 200 and 300 ms, depicting a typical temporal deployment of attention to the cued element as a function of time after the cue.

## S3. Individual Topographies of SSVEP Amplitudes


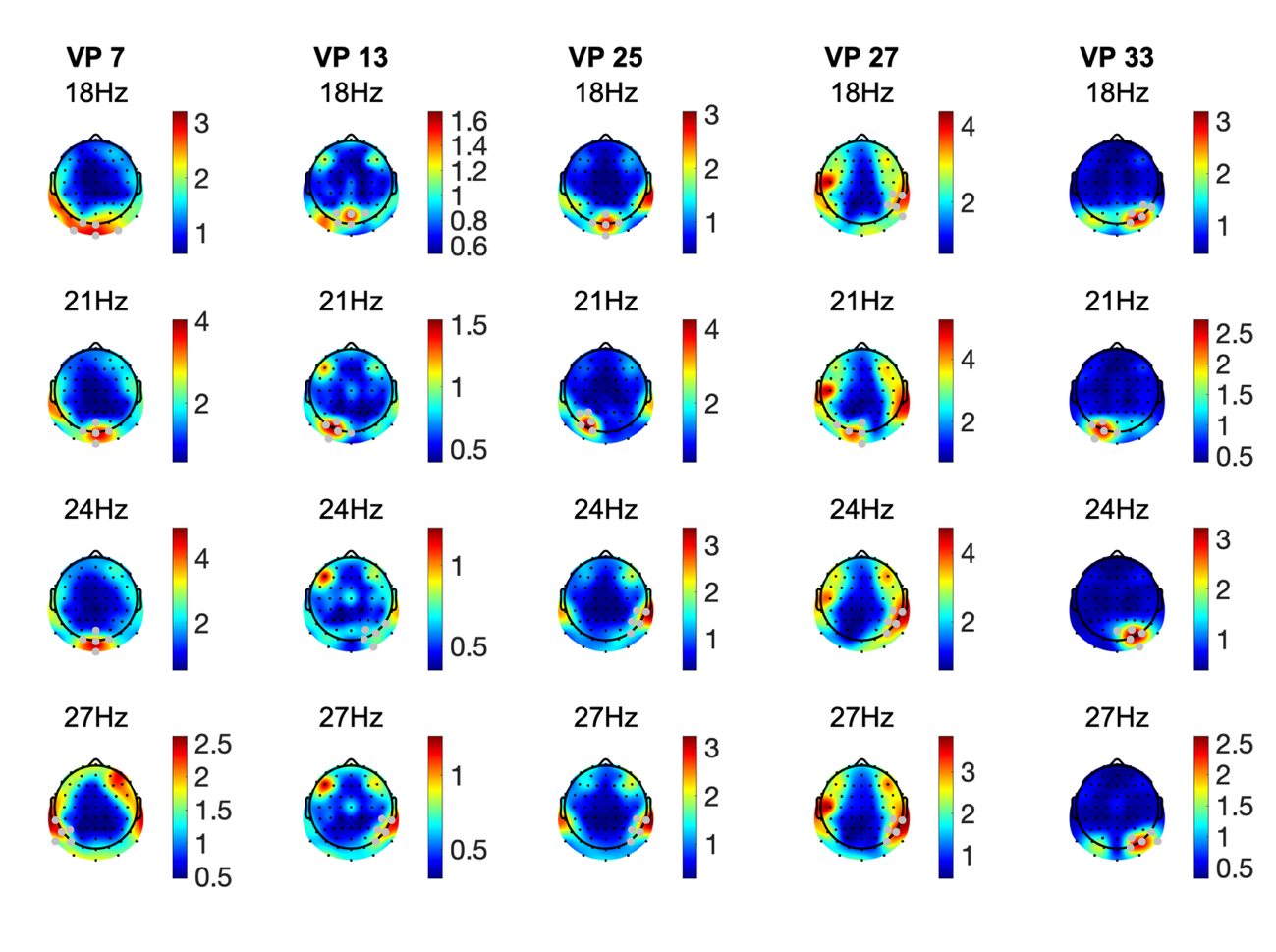


Figure S3: Topographies of SSVEP amplitudes averaged across all conditions in the analysis time window (-1000 to 2000 ms) for a selection of subjects showing individual topographies. Values in colored bars represent µV/cm^2^, and scales are different.

SSVEP amplitude topographies averaged across all conditions and the analysis window (-1000 to 2000 ms relative to the cue) for a selection of subjects that illustrate the mentioned big interindividual variation are depicted in Figure S3. For example, some subjects showed a peak at centrally located electrodes for some frequencies (e.g., Subject 7 and 13, at 18 Hz) but not for other frequencies (e.g., at 27 Hz). For some subjects, the expected lateralization for a flicker presented in the right visual field was not obtained, whereas for stimuli presented in the left hemifield SSVEP amplitudes maximized contralateral to stimulus presentation. Given these topographical variations, a fixed electrode cluster would not be able to represent peak SSVEP amplitudes for all subjects and frequencies. These observations motivated the analysis with an individual electrode cluster based on the individual best electrode.

## S4. Contrast Between Peak- and Sideband SSVEP Amplitudes per Frequency.

Table A1

*Pairwise contrasts comparing peak vs. sideband SSVEP amplitudes across stimulation frequencies (18 Hz, 21 Hz, 24 Hz, 27 Hz). Reported are estimated marginal means (emmeans) contrasts derived from a repeated-measures ANOVA with factors Frequency (Freq: 18–27 Hz) and Band (Peak vs. Sideband). Estimates reflect the amplitude difference (Peak – Sideband), with associated standard error (SE), degrees of freedom (df), t-ratio, and adjusted p-values (Holm correction).*

| contrast | Frequency | estimate | SE | df | t.ratio | p.value |
| --- | --- | --- | --- | --- | --- | --- |
| Peak - Side | 18 Hz | 1.57 | 0.19 | 29 | 8.26 | <.0001 |
| Peak - Side | 21 Hz | 1.67 | 0.18 | 29 | 9.18 | <.0001 |
| Peak - Side | 24 Hz | 1.42 | 0.21 | 29 | 6.84 | <.0001 |
| Peak - Side | 27 Hz | 0.68 | 0.11 | 29 | 6.44 | <.0001 |

## S5. Contrast Between SSVEP Amplitude Enhancement Onset Times for the Uncued Arc Matching in Color with the Cued Arc (*Uncued Match)* and the Uncued Arc Originating From the Different Color Configuration (*Uncued)*.

**
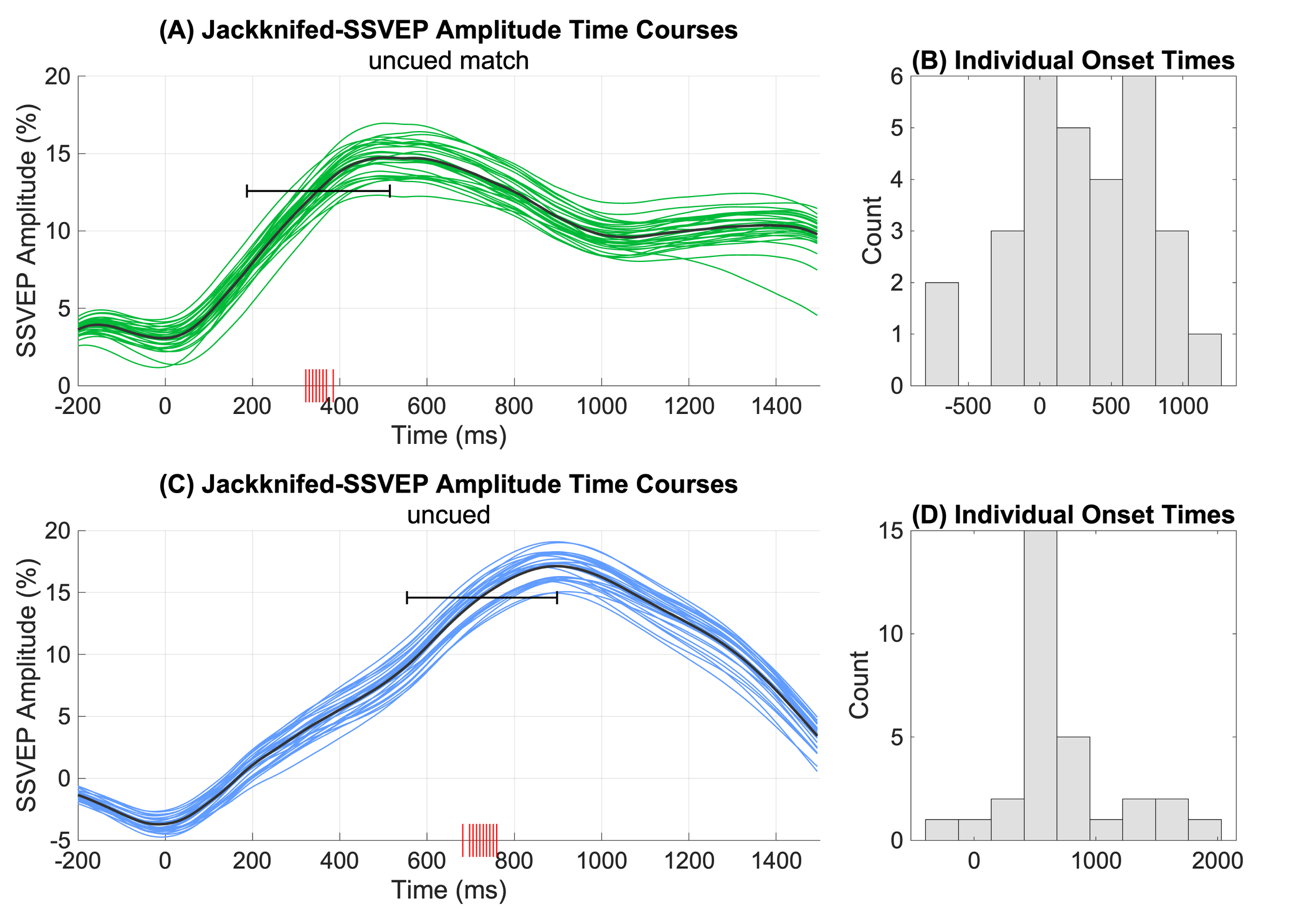
**

Figure S5. SSVEP Amplitude Onset Times. Jackknife-based estimation of SSVEP amplitude onset times for conditions “uncued match” (green) and “uncued” (blue). Panels A and B show results for the uncued match condition, and Panels C and D show results for the uncued condition. The onset times were defined as the time point at which each jackknifed time course reached 85% of its peak value (see Methods for details). **(A)** SSVEP amplitude time courses for individual jackknife samples (light green) and their grand average (black). Red vertical ticks at the x-axis indicate the estimated onset times for each jackknife subsample. The black horizontal error bar represents the mean onset time across corrected individual estimates (Smulders method) ±95% confidence interval. See Methods for details (Section 2.5.3.1 Analysis of Gabor Filtered Time Courses). **(B)** Histogram of Smulders corrected onset times (relative to cue onset), retrieved from jackknife subsamples for the uncued match condition. **(C) and (D)** Same as A and B, but for the uncued condition (blue traces).

**References**

Wickham, H. (2016). *ggplot2: Elegant Graphics for Data Analysis*. Springer New York. https://ggplot2.tidyverse.org
